# Prey Status Affects Paralysis Investment in the Ponerine Ant *Harpegnathos venator*

**DOI:** 10.3390/insects13010026

**Published:** 2021-12-25

**Authors:** Lei Nie, Fei Zhao, Yiming Chen, Qian Xiao, Zhiping Pan, Hao Ran, Yijuan Xu

**Affiliations:** 1 Red Imported Fire Ant Research Center, South China Agricultural University, Guangzhou 510642, China; nielei98@126.com (L.N.); 2020802147@stu.njau.edu.cn (F.Z.); 20213138145@stu.scau.edu.cn (Y.C.); Tiaoo1224@163.com (Q.X.); ranhao.cn@gmail.com (H.R.); 2 Guangdong Laboratory for Lingnan Modern Agriculture, Guangzhou 510642, China; 3 Guangdong Key Laboratory of Animal Conservation and Resource Utilization, Guangdong Public Laboratory of Wild Animal Conservation and Utilization, Institute of Zoology, Guangdong Academy of Sciences, Guangzhou 510260, China; 4 Key Laboratory of Ecology of Rare and Endangered Species and Environmental Protection, Guangxi Normal University, Ministry of Education, Guilin 541004, China

**Keywords:** *Blatta lateralis*, live prey, paralysis behavior, stinging time

## Abstract

**Simple Summary:**

Some ants sting their prey to paralyze or kill them when foraging. Some studies have found that paralysis of prey may also be used in food storage. To further understand the foraging behavior of predatory ants and the underlying mechanism, we systematically observed the foraging behavior of *Harpegnathos venator* when provided with different types of food, different prey sizes, and different prey numbers, and we studied its paralysis behavior in relation to prey under different food supply conditions through controlled experiments. We found that the stings of *Harpegnathos venator* completely paralyzed the cockroaches. The stinging time was significantly longer at a higher prey activity level and for larger cockroaches. In addition, there was no significant difference in the stinging time of *H. venator* for different prey densities. It was much shorter for legless cockroaches than for normal cockroaches. The results showed that the longer the stinging time for similar cockroaches, the longer it took for the prey to revive and move. The results are helpful for further understanding the behavioral mechanism underlying prey storage in predatory insects.

**Abstract:**

The paralysis behavior of some ponerine ants when foraging may be important for food storage and colony development. However, how workers invest in paralysis under different prey circumstances is often overlooked. Here, we report the prey-foraging behavior and paralysis behavior of *Harpegnathos venator* under different food supply conditions. Solitary hunting was the main foraging mode of *H. venator*, with occasional simple collective hunting. Nymphal cockroaches with high activity were the most attractive to *H. venator*. In the experiment, we found that the stings of *H. venator* completely paralyzed the cockroaches. The stinging time was significantly longer at a higher prey activity level and for larger cockroaches. In addition, there was no significant difference in the stinging time of *H. venator* for different prey densities. The results showed that the longer similar cockroaches were stung, the longer it took for them to revive and move. These results are helpful for further understanding the behavioral mechanism underlying the food storage of live prey by predatory insects.

## 1. Introduction

Ants are the most widely distributed and diverse social insects on Earth [1]. They consume a wide range of foods, mainly terrestrial arthropods, the larvae of other ants, plant sap, honeydew secreted by insects such as aphids, seeds, and fungi [2,3]. Although ants consume many kinds of food, food selection may be affected by internal demand and the external environment. For example, the rapid growth of ant colonies increases the demand for high-protein food, which may be obtained by predating other insects [4].

To occupy more food resources, foraging strategy is particularly important. The mechanism of task specialization in most ant species is age polyethism [5]. The older age category of ant workers usually take on the job of foraging, because they are more experienced [3]. While searching for food, ants may encounter a variety of foods and different situations. How to process information and take measures will affect the ant colony’s foraging efficiency. Foragers will collect the characteristics of the food, deciding whether or not to recruit. For large prey, ants will quickly recruit other workers so as to increase the efficiency of foraging [6]. Searching, fighting, and transporting all consume energy. Balancing the pros and cons is very important for an optimal forager [7].

Food resources are unevenly distributed in time and space; therefore, food storage is important, especially for species that consume a single food type. Ants can store food in liquid form through the social stomach (crop) for a short time and then pass it to nest mates through trophallaxis [8]. Food storage by the social stomach is common in Formicinae, Myrmicinae, and Dolichoderinae [9]. In the subfamily Formicinae, the crop of a *Melophorus* and a *Myrmecocystus* worker can be expanded to the size of a pea or even larger, serving as a storage container for sugar, lipid solution, or water [10,11]. Workers of two pollen-feeding tropical ants (*Zacryptocerus texanus* and *Zacryptocerus rohweri*) also use crops to store and transfer pollen to larvae for digestion [12].

Some ants sting their prey to paralyze or kill it when foraging. Studies have found that paralysis of prey may also be used in food storage [3]. Hölldobler [13] found such paralysis-facilitated storage of prey in *Cerapachys* ants (Formicidae; Dorylinae). Living prey larvae (*Pheidole*) can be stored for a long time after the injection of venom. Ponerine ants usually hunt live insects [14]; therefore, the paralyzing effect of the injected toxin can obviously facilitate subduing the prey to ensure hunting success [15]. In addition, the storage of food in a crop is thought to be lacking in Ponerinae ants owing to the morphology of the proventriculus [16], which is probably the reason that some Ponerinae species evolved the way of preserving food by paralyzing. When *Centromyrmex bequaerti* (Formicidae; Ponerinae) are faced with a group of termites, the hunting ants paralyze their prey and arrange them in a pile before returning to the nest [14]. *Harpegnathos saltator* workers (Formicidae; Ponerinae) hunt solitarily and use their sting to paralyze their prey and store prey for a longer time when they prey on cockroaches [17]. Maschwitz, Hahn, and Schnegge [17] also found that *H. saltator* stung large cockroaches for longer periods of time than small cockroaches, which suggests that the supply of prey affects the paralysis behavior of ants. To further understand the foraging behavior of predatory ants and the underlying mechanism, we systematically observed the foraging behavior of *Harpegnathos venator* when provided with different types of food, different prey sizes, and different prey numbers, and we studied its paralysis behavior on prey under different food supply conditions through controlled experiments. The results are helpful for further understanding the behavioral mechanism underlying prey storage in predatory insects.

## 2. Materials and Methods

### 2.1. Insects and Materials

Six *Harpegnathos venator* colonies were collected in Nalong Town, Lingshan County, Qinzhou City, Guangxi Zhuang Autonomous Region (geographic coordinates: 21°51′–22°38′ N, 108°44′–109°35′ E.). Each colony was placed in a plastic box (20 × 12 × 7 cm^3^) with the inner edges coated with a talc powder–ethanol mixture [18]. A plaster nest (a glass-covered plaster tray with a diameter of 8 cm) and a cherry-red cockroach (*Blatta lateralis*) were provided in the plastic box. Each colony contained one queen, 15–30 workers and 4–8 larvae. To study the foraging preference of *H. venator* in terms of food type, we selected certain types of food and arthropods. The cherry-red cockroach (*B. lateralis*), *Tenebrio molitor*, and *Gryllodes sigillatus* were purchased from a local market. The Chinese rice locust (*Oxya chinensis*), *Aporthonia borensis* Hagen, *Achaearanea tepidariorum* Koch, *Odontotermes formosanus*, and *Pseudospiropolellus avernus* were collected on the campus of South China Agricultural University. *Bactrocera dorsalis* Hendel was provided by the Department of Entomology, South China Agricultural University. Pupae of *Solenopsis invicta* Buren were provided by the Red Imported Fire Ant Research Center, Department of Entomology, South China Agricultural University. Watermelon, ham sausage, apple, sugar, biscuits, sesame, raw pork, honey, corn, and vegetables were purchased from local supermarkets. The *H. venator* colonies were maintained at 24 ± 2 °C, 70% RH, and 14:10 h L:D for at least 1 week prior to the experiments. Before all the experiments, the colonies were starved for 24 h.

### 2.2. Observation of the Foraging Behavior of H. venator

The plastic box containing an ant colony was placed inside an observation box (70 × 45 × 18 cm^3^, with a 1 cm layer of clinical gypsum laid on the bottom in advance) with the inner wall coated with a talc powder-ethanol mixture. Five cherry-red cockroaches (nymphs, body length ~1.2 cm) were placed in the observation box, and the small opening reserved at the bottom of the ant feeding box was opened to allow the ants to enter the observation box for foraging. Between 8:00 a.m. and 11:00 a.m., we observed and described the foraging action and mode of the ants with the naked eye. The observation lasted for 30 min, and there were 2 repeated trials with 3 ant colonies.

### 2.3. Prey Preference of H. venator in Terms of Food Type

One cherry-red cockroach and a Chinese rice locust adult as well as fire ant pupae, 10% sugar water, 20% honey water, ham sausage, sweet biscuit, apple slice, watermelon slice, sesame seeds, corn, vegetable leaves, and raw pork of approximately the same weight (50 g) were randomly placed in the observation box. From 6:00 a.m. to 11:00 a.m., a video camera (Nikon FDR-AX100) was used to record and count the frequency of food touching and transport by the ants. To ensure that the data for the different food preferences of the ants were comparable, the experimental data were recorded within 2 h after the first ant entered the foraging area. There were 2 repeated trials with 3 ant colonies.

### 2.4. Prey Preference of H. venator in Terms of Arthropod Species

One medium-sized nymph of *B. lateralis* (body length ~1.2 cm), a medium-sized nymph of *G. sigillatus* (body length ~1.5 cm), a medium-sized larva of *T. molitor* (body length ~1.2 cm), a medium-sized nymph of *O. chinensis* (body length ~1.5 cm), an adult *B. dorsalis* (body length ~0.7 cm), an *A. borensis* worker (body length ~1.2 cm), an adult *A. tepidariorum* (body length ~0.6 cm), and an *O. formosanus* worker (body length ~0.6 cm) were randomly selected and placed in the observation box. Each of these prey items was placed in the foraging area. From 6:00 a.m. to 11:00 a.m., a camera was used to record and count the frequency of prey touching and transport by *H. venator*. The experimental data were recorded within 2 h after the first ant entered the foraging area. There were 2 repeated trials with 3 ant colonies.

### 2.5. Prey Preference of H. venator in Terms of Prey Body Size

One small (nymph, body length ~0.6 cm), one medium-sized (nymph, body length ~1.2 cm), and one large (adult, body length ~2.0 cm) cockroach were selected and placed in the feeding box. From 6:00 a.m. to 11:00 a.m., a camera was used to record and count the frequency of prey touching and transport by *H. venator*. The experimental data were recorded within 2 h after the first ant entered the foraging area. There were 2 repeated trials with 3 ant colonies.

### 2.6. Prey Preference of H. venator in Terms of Insect Activity Level

Small cockroaches (nymphs, body length ~0.6 cm) were selected and treated as follows: (i) active (without any treatment); (ii) clipped (tweezers were used to squeeze their chests to reduce their activity level but allow them to crawl slowly): (iii) newly dead (recently scalded until dead with boiling water); and (iv) 12 h after death (scalded with boiling water and returned to a normal temperature). From 6:00 a.m. to 11:00 a.m., a camera was used to record and count the frequency of prey touching and transport by *H. venator*. The experimental data were recorded within 2 h after the first ant entered the foraging area. There were 2 repeated trials with 3 ant colonies.

### 2.7. Effect of Cockroach Feet Removal on the Stinging Time of H. venator

To further determine the effect of prey activity level on the foraging behavior of *H. venator*, a medium-sized nymphal cockroach (body length ~1.2 cm) was randomly selected. The feet of the nymph were cut off with medical scissors, and the nymph was placed in the observation box. The stinging time of *H. venator* was recorded by video camera. A medium-sized nymphal cockroach with feet served as the control. The experiment was repeated 30 times.

### 2.8. Effects of Prey Density and Body Size on the Stinging Time of H. venator

To determine the effects of prey density on stinging time, two or five medium-sized nymph cockroaches (body length ~1.2 cm) were randomly selected and placed in the observation box. The stinging time of *H. venator* was recorded by video camera. The experiment was repeated using six colonies.

In the experiment on the prey size effect, one small (nymph, body length ~0.6 cm), one medium-sized (nymph, body length ~1.2 cm), or one large (adult, body length ~2.0 cm) cockroach was randomly selected and placed in the observation box. The stinging time of *H. venator* was recorded by video camera. The experiment was repeated with 30 cockroaches for each size treatment.

### 2.9. The Relationship between Stinging Time and the Recovery of Cockroaches

To further study the relationship between stinging time and the recovery of cockroaches, a large adult cockroach (15–17 mm in length) was randomly selected and placed in the observation box. The stinging time of *H. venator* was controlled at 0–9 s or 13–20 s. The recovery of the cockroach was observed every 10 min within 1 h after the sting and every half hour thereafter. This experiment lasted 48 h. The cockroaches were considered awake if they could move forward after being touched with a tweezer. The experiment was repeated 30 times.

### 2.10. Field Experiment

To verify the results of the laboratory experiments, field observations were conducted on Baiyun Mountain in Guangzhou on 7 November 2021.

A medium-sized nymphal cockroach (body length ~1.2 cm) was randomly selected. The feet of the nymph were cut off with medical scissors, and the nymph was placed in the observation box closely connected to the nest of *H. venator* (Figure S1). The stinging time of *H. venator* was recorded by video camera. A medium-sized nymphal cockroach with feet served as the control. There were 3 repeated trials with 3 ant colonies.

In the experiment on the prey size effect, one medium-sized (nymph, body length ~1.2 cm) or one large (adult, body length 2.0 cm) cockroach was randomly selected and placed in the observation box closely connected to the nest of *H. venator* (Figure S1). The stinging time of *H. venator* was recorded by video camera. There were 3 repeated trials with 3 ant colonies.

During the field experiments, newly dead (recently scalded until dead with boiling water) cockroaches were also placed in the observation box to test if they would be attacked by *H. venator.*

### 2.11. Statistical Analysis

SPSS software v. 24.0 was used for the analysis of data. The frequency of touch and transport, the stinging time of *H. venator*, and the recovery time of the cockroaches between treatment groups were compared using generalized linear models (GLMs). The frequency of touch and transport, the stinging time of *H. venator*, and the recovery time of the cockroaches were set as dependent variables; the treatment was set as a fixed effect; and the trial and colony were set as random effects. The Kruskal–Wallis test was used to compare each pair of treatments.

## 3. Results

### 3.1. Foraging Behavior of H. venator

**Figure 1:**
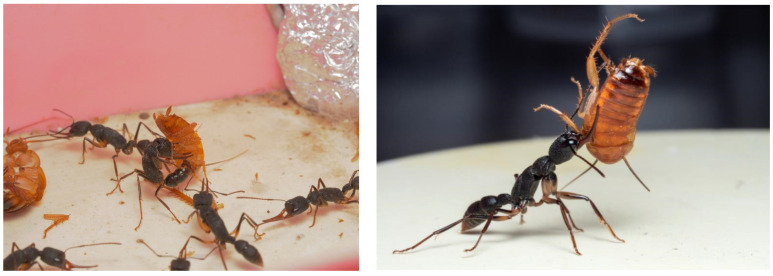
Sting (**left**) and transport (**right**) of *Blatta lateralis* by *H. venator*. Photo by Yanming Liu.

Solitary hunting was the main foraging mode of *H. venator*, but simple collective hunting was occasionally observed during our laboratory and field experiments. Workers were sensitive to moving objects. When an *H. venator* worker observes a moving object, it will exhibit three kinds of behavior. (i) Direct attack behavior. When the worker ant approaches the target, it will initially remain still and quickly shake its abdomen. After a few seconds, it will jump to the prey and clamp it with its upper jaws. After the prey is clamped, the ant will bend its body into a C-shape and stab it with its abdominal stinger. After approximately ten seconds, it will stop using the stinger and bring the clamped prey back to the nest. (ii) When approaching the target, the ant will shake its abdomen, use its antennae to contact the prey, and then attack or leave the target. (iii) The ant will maintain distance from the target, sting and shake its abdomen quickly, and leave after a few seconds (Figure 1).

### 3.2. Foraging Preference of H. venator in Terms of Food Type

**Figure 2:**
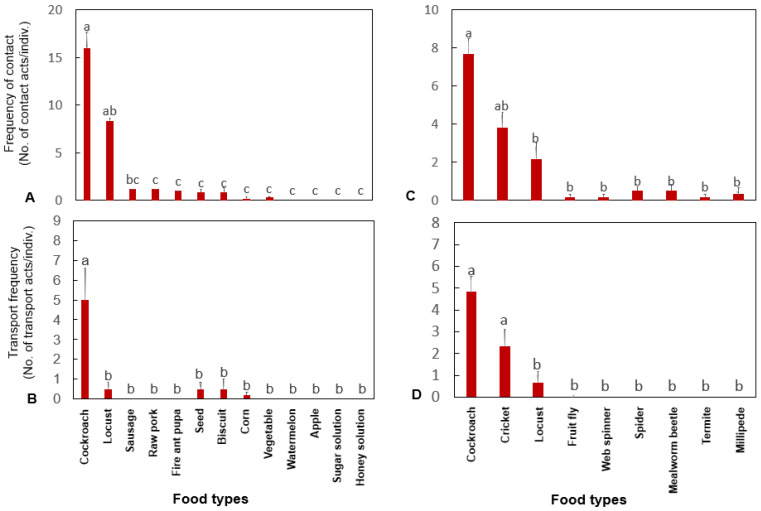
The frequency of touch (**A**,**C**) and transport (**B**,**D**) by *H. venator* for different food types and arthropods (mean ± SE). Bars with the same letter are not significantly different at the 0.05 level (Kruskal–Wallis test).

There were significant differences in the frequency of contact with different kinds of food (Wald *χ*^2^ (12) = 210.312, *p <* 0.0001), and the frequency of contact was significantly higher with cherry-red cockroaches and Chinese rice locusts than with other kinds of food. There was a significant difference in the frequency of transport of different kinds of food by *H. venator* (Wald *χ*^2^ (12) = 44.722, *p <* 0.0001). The frequency of transport was significantly higher for cherry-red cockroaches than for other kinds of food (Figure 2A,B). The results showed significant differences in the frequency of contact with different kinds of arthropods (Wald *χ*^2^ (8) = 92.605, *p <* 0.0001): the frequency of contact was significantly higher for the cherry-red cockroach, the Chinese rice locust, and the white cricket than for other kinds of arthropods. There were significant differences in the frequency of transport by *H. venator* of different kinds of arthropods (Wald *χ*^2^ (8) = 16.137, *p <* 0.0001): the frequency of transport was significantly higher for the cherry-red cockroach and the white cricket than for other kinds of arthropods (Figure 2C,D). No significant difference in the frequency of contact and transport was found among different colonies.

### 3.3. Foraging Preference of H. venator According to Insect Body Size

**Figure 3:**
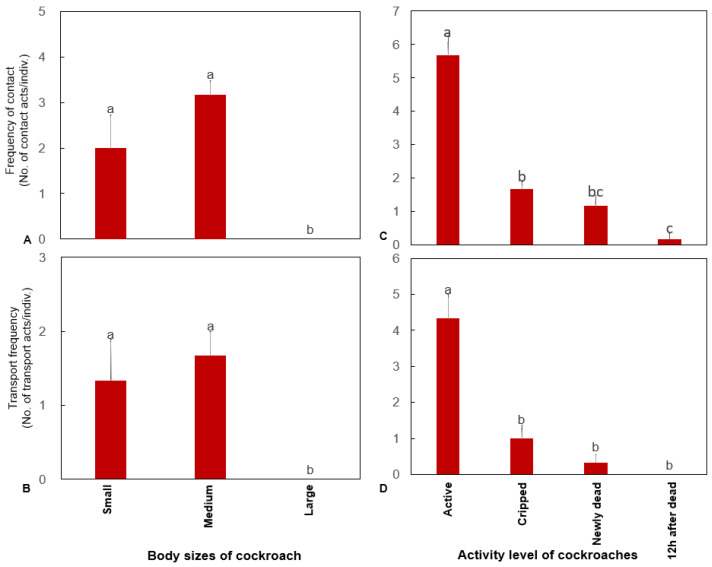
The frequency of touch (**A**,**C**) and transport (**B**,**D**) by *H. venator* for prey with different body sizes and activity levels (mean ± SE). Bars with the same letter are not significantly different at the 0.05 level (Kruskal–Wallis test).

There were significant differences in the contact frequency of *H. venator* with cockroaches of different body sizes (Wald *χ*^2^ (2) = 9.541, *p* = 0.008). The contact frequency was significantly higher with small and medium-sized nymphs than with adults. There were significant differences in the frequency of transport by *H. venator* for cockroaches of different body sizes (Wald *χ*^2^ (2) = 17.00, *p <* 0.0001). The frequency of transport was significantly higher for small and medium-sized nymphs than for adults (Figure 3A,B). No significant difference in the frequency of contact and transport was found among different colonies.

### 3.4. Foraging Preference of H. venator According to Insect Activity Level

There were significant differences in the frequency of contact with cockroaches of different activity levels (Wald *χ*^2^ (3) = 31.056, *p <* 0.0001), and the frequency of contact was significantly higher with active cockroaches than with other cockroaches. There was a significant difference in the frequency of the transport of cockroaches with different activity levels (Wald *χ*^2^ (3) = 20.353, *p <* 0.0001), and the frequency of transport was significantly higher for active cockroaches than for other cockroaches (Figure 3C,D). No significant difference in the frequency of contact and transport was found among different colonies.

### 3.5. Effect of Cockroach Feet Removal on the Stinging Time of H. venator

**Figure 4:**
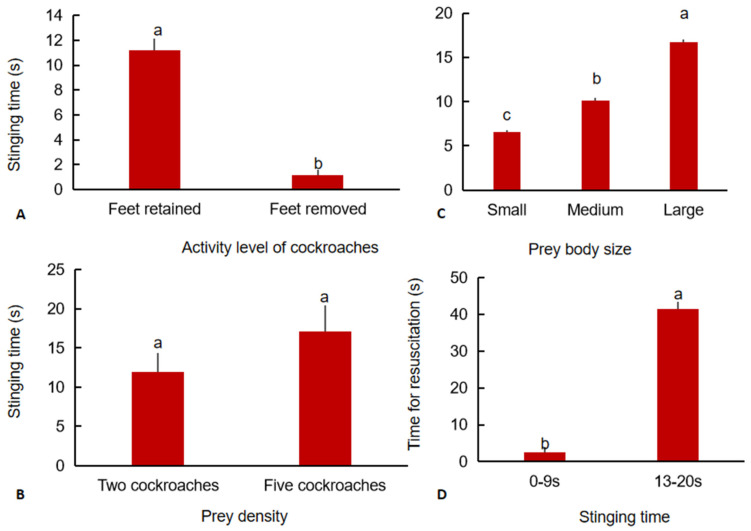
The effect of feet removal (**A**), density (**B**), and body size (**C**) of cockroaches on the stinging time of *H. venator* allocated to a single cockroach and the effect of stinging time on the recovery time of cockroaches (**D**) under laboratory conditions. Bars with the same letter are not significantly different at the 0.05 level (Kruskal–Wallis test).

There was a significant difference in stinging time between the two groups (Wald *χ*^2^ (1) = 45.14, *p <* 0.0001). The stinging time by *H. venator* was significantly lower for the treated cockroaches than for the untreated cockroaches (Figure 4A), which was consistent with the results of the preference test of *H. venator* for insect activity level.

### 3.6. Effects of Prey Density and Prey Body Size on the Stinging Time of H. venator

There was no significant difference in the stinging time of a single cockroach at different prey densities (Wald *χ*^2^ (1) = 0.75, *p* = 0.386) (Figure 4B).

There were significant differences in the stinging time of *H. venator* for cockroaches of different sizes (Wald *χ*^2^ (2) = 59.978, *p <* 0.0001). Specifically, the larger the prey was, the longer the stinging time (Figure 4C).

### 3.7. Effect of Stinging Time on the Recovery of Prey

For cockroaches of the same size, the longer the stinging time, the longer the recovery time. The cockroach recovery time was significantly longer when the stinging time was 13–20 s than when it was 0–9 s (Wald *χ*^2^ (1) = 45.826, *p <* 0.0001) (Figure 4D).

### 3.8. Effects of Feet Removal and Prey Body Size on the Stinging Time of H. venator in the Field

**Figure 5:**
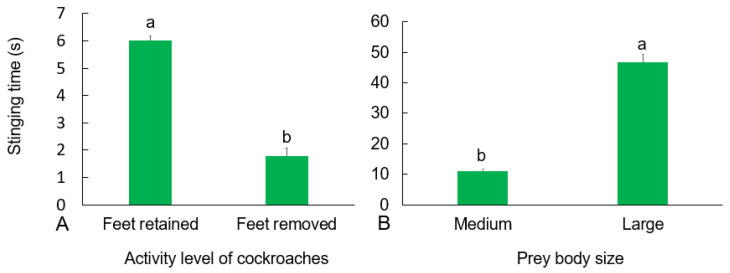
The effect of feet removal (**A**) and size (**B**) of cockroaches on the stinging time of *H. venator* allocated to a single cockroach in the field. Bars with the same letter are not significantly different at the 0.05 level (Kruskal–Wallis test).

The results of the field experiments showed that *H. venator* workers did not attack cockroaches killed by boiling water. The stinging time by *H. venator* workers was significantly shorter for the footless cockroaches than for the untreated cockroaches (Wald *χ*^2^ (1) = 7.962, *p* = 0.005, Figure 5A). Cockroach size also significantly affected the sting time of the worker ants (Wald *χ*^2^ (1) = 6.319, *p* = 0.012, Figure 5B). No significant difference in the frequency of contact and transport were found among different colonies.

## 4. Discussion

### 4.1. Foraging Behavior

The video recordings show that *H. venator* tends to consume arthropods, and the preferred food type is small insects or other arthropods. Other ants of the same genus, such as *H. saltator*, have similar feeding habits [19]. In our experiment, the preferred insect species were cherry-red cockroaches and white crickets. Further research showed that *H. venator* preferred to hunt small and medium-sized cockroach nymphs. It is practicable for *H. venator* to handle large cockroaches, but hunting large cockroaches is not a good choice, probably because the struggle of the large prey will cause the ants to consume more energy. An optimal forager should maximize its overall rate of energy intake [7]. Cockroaches with a high activity were more attractive for *H. venator* to hunt. Similar behavior has been found in other species belonging to the genus *Harpegnathos* [19].

According to direct observations of six colonies, solitary hunting was clearly the main foraging mode of *H. venator*; that is, a single worker ant completed the foraging process alone. Solitary hunting is common for predatory ants. A single worker uses its mandible or sting when hunting small prey [20]. In some cases, worker ants probably chose cooperative hunting. In our research, workers were observed attacking cockroaches simultaneously when they were provided with high-density prey, but under natural conditions, when the prey density was relatively low, they mainly foraged alone [21,22,23].

### 4.2. Paralysis Behavior

The division of tasks among ants changes in a predictable way with age. Older workers tend to do the outside work, such as foraging for food and nest defense. When their venom sacs near fullness, ant workers begin their foraging career [3,24]. Ant venoms are usually used for defense, offense, and communication. Different ants use venoms for different purposes, probably because of the composition of the venoms. The venom of Ponerinae is rich in peptides [25]. As Ponerinae venom has paralytic effects on arthropods, it probably contains neurotoxin peptides that could cause paralysis [26].

The ability of Ponerinae to paralyze prey is likely inherited from their ancestors. For solitary ant hunters, venom is used to capture prey, which is similar to the behavior of the solitary wasp [26]. Wasps use a neurotoxin to paralyze their prey to varying degrees, after which they will inject venom to anesthetize the prey, return to the nest to lay eggs, and then parasitize the prey to meet the nutritional needs of the developing larvae [27]. For example, *Ampulex compressa* (Sphecidae) hunts adult *Periplaneta americana* and then feeds the hatched larvae by injecting venom into the head of the cockroach to paralyze the nervous system, bringing the prey back to the nest and laying eggs in its abdomen [28]. The venom of *Eupelmus orientalis* (Eupelmidae) can cause permanent anesthesia in prey [29]. The venom of *Philanthus triangulum* (Crabronidae) can anesthetize the desert locust (*Schistocerca gregaria*) [30]. *Bracon hebetor* (Braconidae) can permanently anesthetize and parasitize *Galleria mellonella* [31]. The venom of the parasitoid *Pimpla hypochondriaca* (Ichneumonidae) can place adults of the tomato armyworm *Lacanobia oleracea* under permanent anesthesia [32].

*H. venator* prefers active prey, and paralysis can quickly subdue the prey and make it easy to move back to the nest. In the experiment, we found that the stings of *H. venator* paralyzed the cockroaches and rendered them unable to move. The results showed that the longer the similar cockroaches were stung, the longer it took them to revive and move, which explains why *H. venator* workers sting larger cockroaches for longer periods of time than smaller ones (with the assumption that more venom is injected) [17]. This finding further suggests that *H. venator* workers control the duration of stinging to ensure that the cockroaches do not awaken quickly and crawl away. The results show that the abundance and body size of the prey and the physical condition and movement speed of the predator and prey determine the specific mode of predation [33,34,35,36,37]. In addition, the stinging time of *H. venator* was much shorter for legless cockroaches than for normal cockroaches under both laboratory and field conditions, which showed that the ants were more interested in active prey. When an ant encounters a legless cockroach, it does not sting, or the stinging time is very short, which indicates that the ant judges the activity level of the prey to determine the stinging time. In our experiment, we found that the control cockroaches were stung longer and needed a longer time for revival than the legless cockroaches of the same size, indicating that ants may control the degree of anesthesia and preservation of prey through stinging time. This further shows that ants can control the stinging time according to the status of their prey.

In conclusion, *H. venator* prefers to feed on more active insects, and the workers mainly hunt them alone. To achieve the best anesthetic and preservation effect, the stinging time can be controlled according to the prey status. The results provide a new perspective for further understanding the mechanisms underlying ant foraging and food storage behavior. In addition, the sample sizes in this study were relatively small for the experimental behavioral studies, and more observations are required in the future.

## Data Availability

The data presented in this study are available on request from the correspondence author.

## Supplementary Materials

The following supporting information can be downloaded at: https://www.mdpi.com/article/10.3390/insects13010026/s1, Figure S1 Field experiment for the paralysis behavior of *Harpegnathos venator* when provided with different types of preys.
