# Supplementary material for: Prey Status Affects Paralysis Investment in the Ponerine Ant Harpegnathos venator
Source: Insects. 2021 Dec 25;13(1):26. doi: 10.3390/insects13010026 (PMC8780582; doi:10.3390/insects13010026)
Supplement: Supplementary file 1 [file insects-13-00026-s001.zip › insects-1494511-SI.pdf]

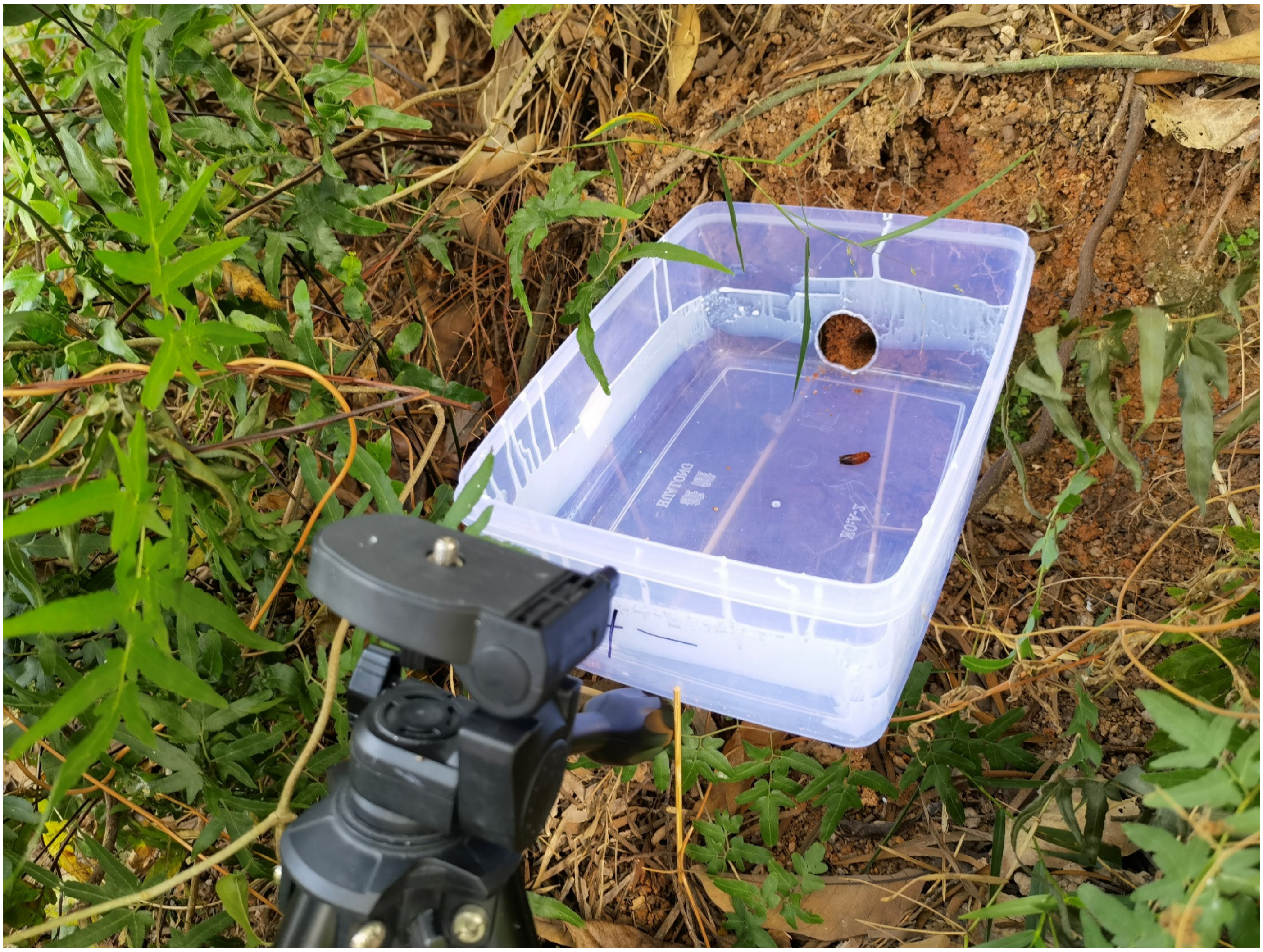

Figure S1. Field experiment for the paralysis behavior of Harpegnathos venator when provided with different types of preys.
